# Combining stable isotope and WQI methods to study the groundwater quality: a case study in Essaouira city, Morocco

**DOI:** 10.1007/s42452-022-05165-y

**Published:** 2022-10-23

**Authors:** Mohammed Bahir, Otman El Mountassir, Driss Dhiba, Abdelghani Chehbouni, Paula M. Carreira, Hakam elbiar

**Affiliations:** 1 High Energy and Astrophysics Laboratory, Faculty of Sciences Semlalia, Cadi Ayyad University, P.O. Box 2390, 40000 Marrakesh, Morocco; 2 Office Chérifien des Phosphates (OCP), Ben Guerir, Morocco; 3 Institut de Recherche Pour le Développement, Unité Mixte de Recherche, Centre D’études Spatiales de La Biosphère, 31401 Toulouse, France; 4 Centro de Ciéncias e Tecnologias Nucleares, C2TN/IST, Lisboa University, Lisbon, Portugal

**Keywords:** Essaouira Basin, IWQI, WQI, Groundwater quality, COVID-19, Stable isotope

## Abstract

Groundwater is an important water resource in arid and semi-arid regions. Therefore, this study aimed to assess groundwater's suitability for drinking and irrigation using the Water Quality Index (WQI) and the Irrigation Water Quality Index (IWQI). To this end, groundwater data were collected from 58 sites in 2019 (wet season) and 61 samples in 2020 (dry season) in the Meskala-Ouazzi sub-basin. The Piper diagram showed that Ca–Mg–Cl was the dominant groundwater facies type. The confinement due to COVID-19 has significantly improved the water quality of the Meskala-Ouazzi sub-basin. Instead, approximately 50% of sites showed improved water quality when calculating the WQI and IWQI. However, the sodium adsorption ratio (SAR) showed that most samples below 10 are found in all of the examined samples, which are mostly found, indicating excellent irrigation water, and the Wilcox diagram depicted 20.14% of samples lying in the unsuitable region. Stable isotopes (δ^18^O and δ^2^H) of groundwater reveal that local precipitation is the main source of recharge. However, groundwater recharge is affected by the evaporation process due to the different geological conditions caused by topographic differences in the study area. The present study is useful for proper planning and managing water resources available for consumption and irrigation.

## Highlights

- Water quality has improved during lockdown due to COVID-19.
- Assessment of water drinking and irrigation by two models (WQI, and IWQI) in a semi-arid environment.
- The main source of groundwater recharge in coastal aquifers is rainfall, based on a stable isotope (δ^2^H, δ^18^O).

## Introduction

Groundwater is an indispensable part of human living space and the hydrological cycle, providing high-quality freshwater resources for human beings. It is important for global domestic, industrial, and agricultural use [1]. Furthermore, water is the foundation of life, a necessary input for social and economic development, and a key element of environmental sustainability [2–4]. One of the greatest pressures on freshwater resources in agriculture is food production. Agriculture is responsible for nearly 80% of global freshwater withdrawals when all factors are included (up to 90% in some rapidly growing economies) [2].

Today, the quantity and quality of groundwater have become critical issues for many places [5, 6]. Indeed, rapid population growth and socio-economic development have increased water resource demand. These actions would have had several long-term impacts on groundwater resources, including a general drop in water levels, an increased likelihood of saltwater intrusion, and degradation of water quality [2]. Groundwater pollution in arid and semi-arid areas can have a wide range of environmental, social, and economic effects and cause health and livestock problems [5].

Apart from its consumptive and domestic functions, groundwater is a major natural gift that contributes to the expansion of any country's agriculture, industrialization, and socio-economic development [2–4]. The chemical characteristics present and their concentration, mainly obtained by geological and other anthropogenic processes in a given region, determine groundwater quality [2–4]. The rapid population development, industrialization, agricultural pesticides, and disposal of urban and industrial waste have all played key roles in groundwater contamination, putting immense pressure on water resources [2–4].

In the Essaouira basin, several studies have already been carried out. Based on the application of geochemical and isotopic techniques, these investigations have discovered and defined some of the main factors responsible for water quality degradation in this region's particular coastal areas [7, 8]. These investigations have shown that runoff and precipitation contribute to groundwater recharge in the Essaouira basin, (ii) that water–rock interaction plays an important role in groundwater mineralization, and (iii) that anthropogenic pollution has been detected [7, 8]. The novel coronavirus (COVID-19) began to spread worldwide towards the end of 2019 [9, 10]. With infections across 210 countries and a rising number of deaths, the World Health Organization (WHO) declared the COVID-19 outbreak a global pandemic and health emergency [9, 10]. As of April 3, 2022, 493,673,607 confirmed cases of COVID-19 worldwide have been reported to WHO, including 6,169,931 deaths (WHO COVID-19 Dashboard). Due to the direct and indirect effects of the COVID-19 pandemic, there has been a noticeable environmental recovery. According to satellite data, significant NO_2_ (nitrogen dioxide) reductions have been observed in major cities in China, Europe, the United States, and India [9, 10]. Many researchers [9–11] reported on the influence of COVID-19 on water resources, and Krishan and Khan [10, 11] reported on its impact during and after COVID-19. In recent years, groundwater quality assessment and spatial analysis based on combining Geographic Information Systems (GIS) with WQI methods have proven to be a powerful tool for spatial information management of groundwater resources [12–14]. The WQI is an efficient tool to access water quality and its suitability for drinking. It was first developed by Horton and Brown [13, 14] and has been widely used in numerous water quality assessment studies.

The main objective of this study was to examine the favorable influence of closure on environmental indicators such as water quality in the Essaouira region (Morocco). This was a unique opportunity to assess the impact of reduced human activity on groundwater quality. This period was separated into a pre-closure phase (March 2019) and a post-closure phase (July 2020). According to several research studies [9–11], containment has become a viable remedial technique to improve the quality of various environmental resources.

The objective of this study is to:

1. Assess the suitability of the groundwater for human consumption and irrigation purposes by using two methods WQI and IWQI.
2. Map the spatial distribution of the groundwater quality (WQI and IWQI) between two years 2019 and 2020.
3. Determine recharge sources and groundwater dynamics using stable isotopes (δ^18^O and δ^2^H) before and during confinement due to the COVID-19 pandemic in the Essaouira region.

The conclusions of this study are important for the long-term exploitation and management of groundwater in the Essaouira basin.

## Study area

### Location and climate

**Fig. 1:**
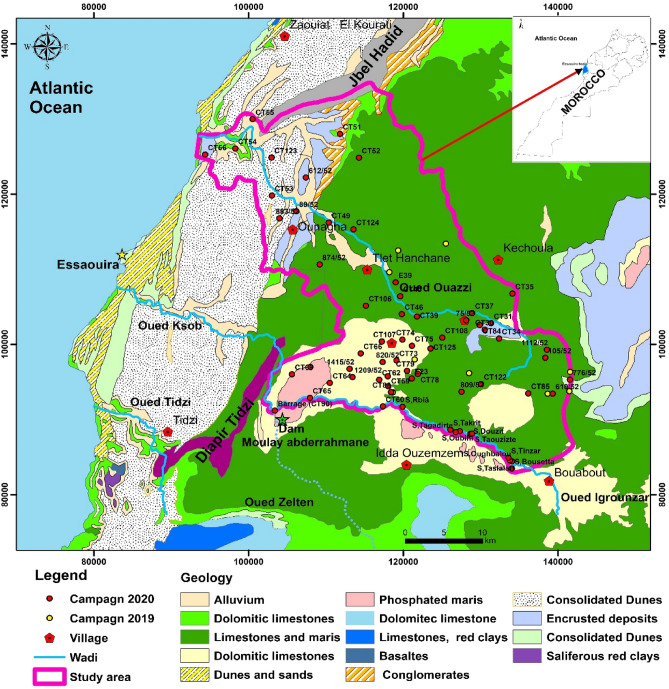
Map of the study area, showing sampling locations and geological

The geographical situation of the city of Essaouira is located 100 km south of the city of Safi and 130 km north of the city of Agadir in the extreme western part of the High Atlas. It extends into a coastal area largely open to the Atlantic Ocean, with a rectilinear shape with a general NNE-SSW direction (Fig. 1). The Meskala-Ouazzi sub-basin has an average elevation of 382 m, with the lowest and the highest elevations of 4 m and 714 m, respectively. Two rivers cross the Meskala-Ouazzi sub-basin: Wadi Ouazzi and Wadi Igrounzar, whose natural outlet is the Atlantic Ocean [15–17].

The study area was selected to assess the groundwater quality of the Meskala-Ouazzi sub-basin, which is located in the Essaouira basin in the southwestern region of Morocco (Fig. 1). It is bounded to the north by the Jbel Hadid anticline, to the south by the Oued Igrounzar, to the east by the Bouabout region, and to the west by the Atlantic Ocean; the present study area covers an area of 1184 km^2^ (Fig. 1). The Essaouira basin has a semi-arid climate with an annual rainfall of about 300 mm and temperatures between 20 and 30 degrees Celsius [15–17]. Four hundred thousand people live in this predominantly rural region. The area of the Essaouira basin is 6000 km^2^ and is characterized by scarce and irregular water resources [15–17].

### Geological and hydrogeological settings

On the geological plan, the upstream part of the study area (east) is dominated by the middle and upper Cretaceous outcrops, particularly Albian-Vraconian, Cenomanian, and Turonian deposits, which dominate the study area geologically [18]. These formations consist of banks of limestone and dolomitic intercalated with marls and sandstones. The Albian and Vraconian are represented by green marls (with a thickness of 150 m) and dolomitic limestones (with a thickness of 140 m). In modest quantities, an alternating series of gray marls with laminated anhydrite, lumachellic limestones (0–15 m), dolomitic limestones, and sandstones characterize the Cenomanian. It has an average thickness of 200 m [19]. The Turonian consists of limestones, where silica is very abundant [20]. The outcrop of the Plio-Quaternary formations characterizes the downstream part of the study area (west) [18–20]. These are represented by conglomerates, alluviums, colluviums, and sandstones (Fig. 1).

The study area has two main aquifer systems: (1) a multi-layer aquifer made primarily of detrital deposits (sandstones, conglomerates, and sands) from the Plio-Quaternary aquifer, which supplies the majority of the water to the population; and This has direct contact with the Triassic and Cretaceous strata underneath; and (2) Cenomanian–Turonian calcodolomitic layers, which Rachid et al.[18] claim indicate a karstic aquifer. Between 400 and 700 m above sea level (a.s.l.) on the Jbel Kchoula, the Turonian horizon disappears, while the Plio-Quaternary layers disappear between the sea level and 400 m a.s.l [18–20].

The transmissivity levels are approximately 4.5 × 10^–5^ to 6 × 10^–2^ m/s, while the hydraulic permeability is 3.2 × 10^–2^ m/s in the study area [19, 20].

## Materials and method

### Sample collection and analysis

In the study area, 58 groundwater samples were collected before COVID (March 2019) and 61 samples during the COVID period (July 2020) according to standard procedures of the American Public Health Association [21]. The positions of the sampling sites were recorded using a portable GPS. To ensure the reliability of the groundwater sampled, the wells were pumped for at least 5 min until the chemical conditions of the groundwater were stable. Before sampling, all samples were collected in disposable polyethylene bottles that had been washed and rinsed 3–5 times. A 200 m piezometric probe was used to assess the water depth in the wells. To overcome the environmental impact, all physical parameters, such as temperature, pH, and electrical conductivity (EC), were immediately measured in the field with portable multiparameter equipment (Hanna HI9829). Great care was taken when transporting the groundwater samples to the laboratory to preserve their chemical characteristics.

The physico-chemical parameter analyses of the water samples taken during the 2019 campaign were carried out at the Ecole Normale Supérieure (ENS) Laboratory of Geosciences and the Environment in Marrakech city (Morocco country). The nephelometric approach was used to determine the sulfate concentration (SO_4_). The calcium (Ca) concentration was determined using the complexometric method (EDTA), while the chloride (Cl) and magnesium (Mg) concentrations were determined using the Mohr method. Flame photometry was used to determine sodium (Na) and potassium (K) concentrations [21]. Titration with a sulfuric acid solution was used to determine bicarbonate (HCO_3_) concentration [21]. For the 2020 campaign, the chemical analyses were conducted in Mohamed VI Polytechnic University of Benguerir-UM6P-(Morocco) labs. The samples analyses for Ca, Mg, Cl, SO_4_, NO_3_ and HCO_3_ were performed using a SKALAR San++ Continuous Flow Analyzer (CFA), while Na and K concentrations were measured using Atomic Absorption Spectrophotometry (AAS).

### ***Stable isotope data (δ***^***18***^***O and δ***^***2***^***H) analysis***

The refrigerated water samples collected for stable isotopes (δ^18^O and δ^2^H) were analyzed at C^2^TN/IST (Portugal) laboratories, using the mass spectrometer SIRA 10 VG-ISOGAS for δ^2^H and δ^18^O determinations. To improve analytical precision, each sample was measured three times using the procedures proposed by Friedman [22] and Epstein and Mayeda [23] for δ^2^H and δ^18^O, respectively. The water samples were equilibrated with CO_2_ and H_2_ to quantities of δ^18^O and δ^2^H values, respectively, using the standard method [21]. Then, the instrument was calibrated to determine the δ^18^O and δ^2^H composition by analyzing IAEA standards, i.e., Vienna standard mean ocean water (VSMOW) with a precision range ± 1.0‰ for δ^2^H and ± 0.1‰ for δ^18^O. The results of the isotopes are expressed in terms of per mil (‰) relative to VSMOW using the ‘δ’ notation and Eq. (1).

1 $$\delta \;\left( \permille \right)\; = \;\left( {\left( {R_{{{\text{sample}}}} \; - \;R_{{{\text{standard}}}} } \right)/R_{{{\text{standard}}}} } \right)\; \times \;100$$

here R_sample_ is the ratio of δ^18^O/ δ^16^O and ^2^H/H isotopes for the collected groundwater sample, and R_standard_ is the ratio of δ^18^O/ δ^16^O and ^2^H/H isotopes for the standard water sample. The reference standard is usually considered IAEA VSMOW, and the measurement precision is ± 0.1‰ and ± 1‰ for δ^18^O and δ^2^H, respectively. The isotope data reported in this paper correspond to VSMOW.

### Quality analysis of hydrochemical data

For quality control and analytical accuracy, the concentrations of total cations and total anions of each sample were recalculated from (mg/L) to (mEq/L), and the ionic equilibrium error was calculated using Eq. (2), considering those samples that were within the acceptable limit of ± 10% [24].

2 $${\text{IB}}\; = \;100\; \times \;\frac{{\sum {\text{Cations}}\; - \;\sum {\text{Anions}}}}{{\sum {\text{Cations}} \; + \;\sum {\text{Anions}}}}$$

Within the ± 10 range, all analyzed samples were balanced.

### Water quality

Water quality for drinking use will be assessed using the water quality index (WQI) method. WQI is an index that reflects the composite impact of various water quality parameters [17–19]. Water quality explains the physical and chemical properties of water. Various water quality indicators (WQI) have been developed to monitor and analyze the quality of freshwater fit for human consumption. Furthermore, WQI is a well-defined and complicated method for expressing groundwater quality in a single number by combining the values of various physicochemical parameters [25]. Each of the eleven parameters was assigned a weight (w_i_) according to their relative importance in the overall quality of water for drinking purposes [25–28] (Table 1). The maximum weight of five was assigned to a parameter TDS and NO_3_ because of its major importance in water quality assessment. A minimum weight of one was assigned to those parameters deemed insignificant to the overall water quality like Mg. Other parameters were assigned weights between 1 and 5 based on their relative significance in the water quality evaluation [26]. The following equations (Eq. 3) and (Eq. 4) were used to calculate the water quality index using the weighted arithmetic index technique.

3 $$WQI = \sum {\left[ {W_{i} \times \left(\frac{{C_{i} }}{{S_{i} }}\right) \times 100} \right]}$$

4 $$W_{i} = \frac{{\omega_{i} }}{{\sum {\omega_{i} } }}$$

**Table 1:** Groundwater standards World Health Organization (WHO)

Physico-Chemical parameters	WHO Standard (2011. 2017)	Weight (wi)	Relative weight (Wi)
pH	6.5–8.5	4	0.114
EC (µS/cm)	1500	4	0.114
TDS (mg/L)	600	5	0.143
Cl (mg/L)	250	3	0.086
SO_4_ (mg/L)	250	4	0.114
NO_3_ (mg/L)	45	5	0.143
HCO_3_ (mg/L)	120	3	0.086
Na (mg/L)	200	2	0.057
Ca (mg/L)	75	2	0.057
Mg (mg/L)	50	1	0.029
K (mg/L)	12	2	0.057
		35	1.000

WQI levels are categorized as follows: 0–50, excellent; 50–100, good; 100–200, poor; 200–300, extremely poor; and > 300, unfit for drinking [14].

To evaluate water quality for irrigation purposes, the Wilcox diagram was used [29], which uses percent sodium (Na%) obtained by Eq. (5), classifying the water into five categories (excellent, good, acceptable, ordinary, and inadequate).

5 $$Na\% = \frac{{Na^{ + } + K^{ + } }}{{Ca^{2 + } + Mg^{2 + } + Na^{ + } + K^{ + } }} \times 100$$

The SAR is an important irrigation quality index, which also evaluates the contents of cations expressed in milliequivalents per liter [30] according to Eq. (5):

6 $$SAR = \frac{Na + }{{\sqrt{\frac{1}{2}} (Ca^{2 + } + Mg^{2 + } )}}meq/L$$

### Geospatial analysis

The spatial distribution of the WQI and IWQI was carried out using the Inverse Distance Weighting (IDW) interpolation method of the spatial analysis tool in ArcGIS 10.2.2 software. IDW was chosen over Kriging and other interpolation methods as the region is almost homogenous with less irregularity in the measured and calculated values of the parameters and indices. IDW is a deterministic approach to determining the anticipated value by averaging all known locations and giving neighboring spots more weight.

## Results and discussions

### Water-table monitoring

**Fig. 2:**
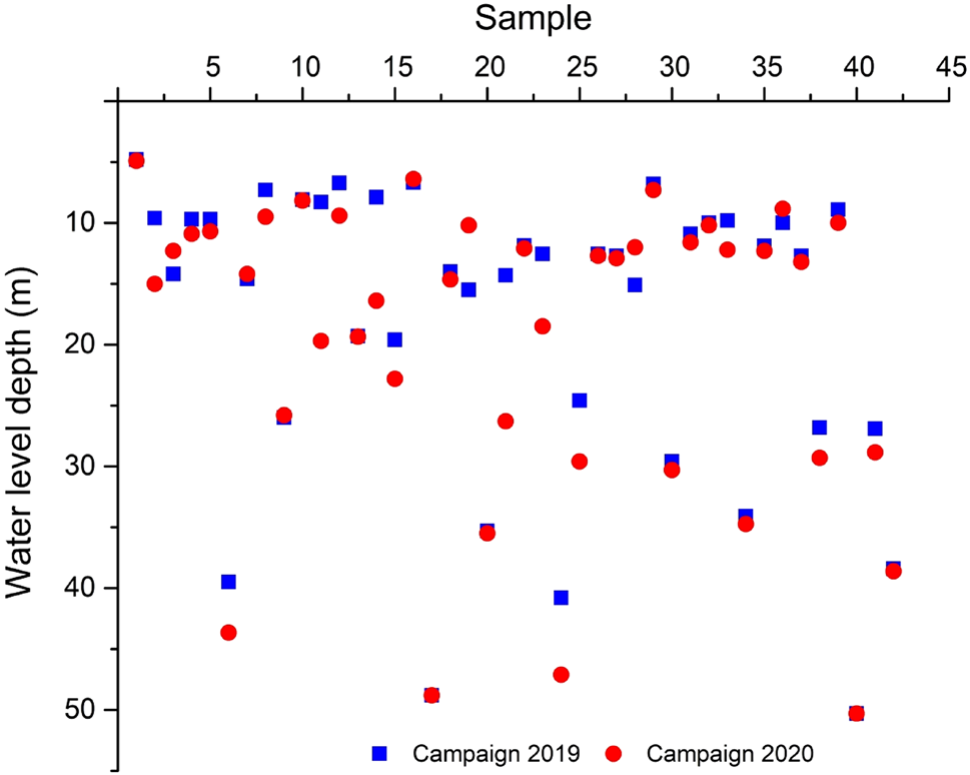
Differences in water-level depth in piezometers, comparing March 2019 and July 2020

Water-table monitoring and piezometric determinations show a slight water level rise of more than 2 m between March 2019 and July 2020 (Fig. 2). The groundwater piezometric investigation in the Meskala-Ouazzi sub-basin revealed the groundwater flow direction, recharge zones, and linkage between these two aquifer layers for shallow (downstream part) and deep aquifers (upstream part). The piezometric analysis of the studied region revealed that the waters of the Meskala-Ouazzi sub-basin flow from northeast to northwest and northeast to southwest, respectively.

For the 2019 campaign, piezometric levels will range from 4.8 to 50.3 m, and for the 2020 campaign, piezometric levels will range from 4.9 to 53.8 m (Fig. 2). The main recharge zones may be found around the Bouabout and Maskala regions in the upstream section of the research area, resulting in a mainly NE–NW groundwater flow direction from upstream to downstream of the sub-basin, and eventually to the Atlantic Sea.

### Hydrochemical characterization and water type

**Fig. 3:**
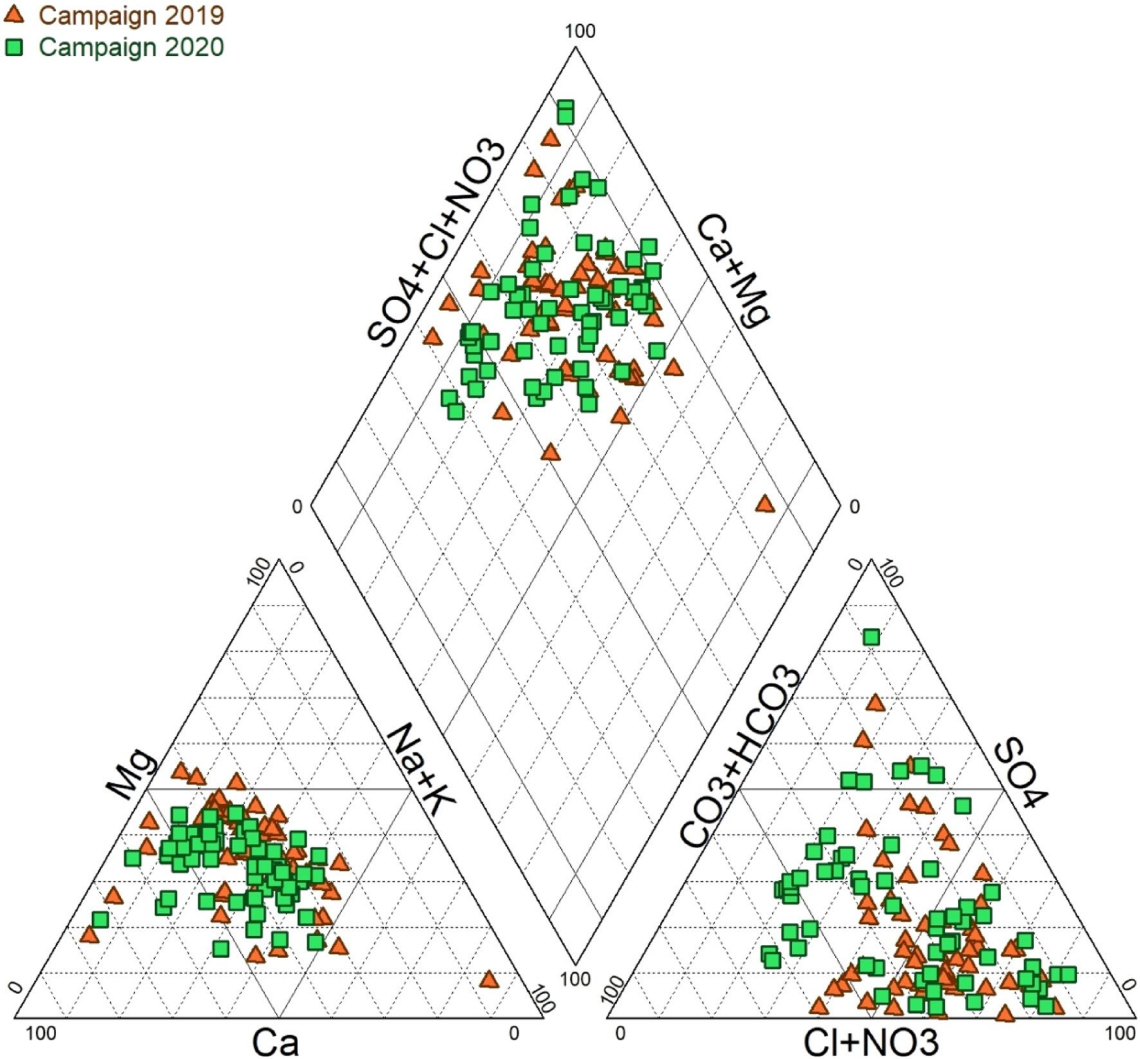
Piper diagram of groundwater samples in the study area

In this study, the trilinear Piper diagram [31] (Fig. 3) was used in identifying the groundwater types, which revealed that the mixed Ca–Mg–Cl type of water is the dominant facies for the campaigns in 2019 and 2020, respectively, in the study area.

### Groundwater quality for irrigation

The relatively high concentration of one ion compared to the other has a negative impact on soil, water and ultimately plants. Therefore, the present study analyzes the quality of groundwater in the region regarding various indices such as electrical conductivity (EC), sodium adsorption ratio (SAR), percent sodium (%Na), and IWQI.

#### Electrical conductivity

The groundwater in the study area is moderately to highly salinized. For the 2019 campaign, electrical conductivity (EC) ranges from 615 to 5738 μS/cm, with an average value of 4479 μS/cm (Table 2). Electrical conductivity (EC) ranges from 541 to 4890 μS/cm for the 2020 campaign, with an average value of 2061 μS/cm. The decrease in salinity between 2019 and 2020 indicates an improvement in water quality.

**Table 2:** Chemical composition of analysed samples of the campaign 2019 and 2020

Sample	pH	T (°C)	EC (µS/cm)	TDS (mg/L)	Ca (mg/L)	Mg (mg/L)	Na (mg/L)	K (mg/L)	HCO_3_	Cl	SO_4_	NO_3_	IB (%)
Campaign 2019													
L1	7.44	21.48	4530	2266	293.4	230.1	395	43.3	497.8	1207	945	30.5	− 9.2
L2	7.5	19.21	2128	1059	139.5	105	180.5	19.2	595.4	581.6	15.6	7	− 5.3
L3	7.82	18.02	2249	1125	152.3	96.5	197.4	10.3	373.4	567.4	268.5	60	− 8.1
L4	7.45	19.71	4200	2105	43.3	34	671.1	11.5	353.8	1178.6	45	30	− 8.0
L5	7.13	21.71	2179	1090	188.7	141.5	125.7	9.6	573.4	639	95	54	− 6.1
L6	8.38	17.8	615	308	82	41.1	32.6	5.1	244	185.5	39.1	4.5	− 5.7
L7	7.71	20.75	2381	1190	181.2	102.1	130.8	8.6	597.8	468	229.1	12	− 9.0
L8	7.45	20.18	3842	1924	365.5	99.1	225.8	15.2	424.6	646.2	812.6	14	− 7.2
L9	7.55	20.27	2199	1099	262.9	89.9	159.3	7.8	358.8	539.6	283.2	8	0.9
L10	7.2	23.63	1075	538	118.6	95.9	27.3	2.5	488	326.6	18.5	18	− 8.6
L11	7.21	24	972	486	102.6	62.2	153	2.1	488	213	27.4	108	1.9
L12	7.51	22	1888	946	134.7	98	120.6	4.6	424.6	454.4	112.6	30	− 5.7
L13	7.45	21.06	4380	2192	430.3	74.8	394.6	12.6	524.6	1349	177.4	120	− 7.4
L14	7.66	21.35	2500	1259	150.2	93.7	292.9	14.6	475.8	852	12.6	7	− 6.4
L15	7.6	20.9	3016	1515	168.3	72.9	285.3	19.6	488	681.6	97.9	160	− 7.7
L16	7.78	19.96	736	368	140	56	18.1	1.5	290.4	188.8	35	24	5.2
L17	7.55	18.91	882	441	181	88.9	14.9	3.4	549	241.4	59.7	18	− 0.8
L18	7.76	20.95	796	398	103.4	80.7	12.4	1.6	500.2	184.6	15.6	13	− 5.9
L19	7.45	21.44	1510	753	160.3	110.5	81.4	2.7	488	411.8	150.9	19.5	− 5.4
L20	7.42	20.8	1559	778	132.2	95.8	83	3	424.6	468.6	80.8	12	− 9.7
L21	7.81	19.6	2123	1061	264.5	140	130.5	5.5	719.8	624.8	77.4	15	− 1.2
L22	7.08	19.45	3690	1848	258.1	165	299	5	573.4	1150.2	177.4	84	− 8.4
L23	7.16	20.3	5738	2867	484.2	102.1	540.6	11.7	561.2	1817.6	268.5	70	− 8.8
L24	7.78	21.26	1020	510	112.2	81.1	44.2	4	485.6	227.2	74.4	21.5	− 6.4
L25	7.76	19.3	1336	668	135.8	95.1	65.3	9.8	436.8	298.2	262.6	17.5	− 9.3
L26	7.99	18	2750	1374	269.3	167.5	98.9	12.5	378.2	454.4	819.1	64	− 7.6
L27	7.47	21.4	1411	704	134.7	100.8	75	3.3	500.2	269.8	256.8	14	− 7.6
L28	7.4	19.63	1584	793	157.1	110.6	100.5	5.3	475.8	312.4	418.5	5	− 8.4
L29	7.3	18.8	2173	1088	232.5	156.5	90.1	5.8	561.2	284	595.6	37.5	− 2.8
L30	7.98	14.85	1843	922	145.9	91.2	100	21.6	475.8	411.8	77.4	78	− 6.2
L31	7.08	16.5	1574	787	102.6	91	81.2	5	410	300.8	128.5	30	− 6.1
L32	7.57	20.3	1543	972	182.8	175.9	96.3	16.8	434.4	369.2	383.2	14	4.6
L33	7.09	17.25	3646	1824	769.5	108.9	55.8	9.9	536.8	340.8	1942.1	12	− 8.2
L34	7.59	18.04	3389	1710	420	260.5	151.7	11.4	683.2	667.4	1295	40.5	− 7.8
L35	7.53	17.5	1192	596	134.7	67.2	79.6	4.9	380.2	312.4	103.8	14	− 4.8
L36	7.2	22.15	1261	631	125	97.3	55.8	2.8	400.2	298.2	215.6	24	− 8.5
L37	7.8	21.03	862	431	88.2	31.7	56.4	0.8	302.6	113	27.9	30	1.4
L38	7.24	20.5	4965	2487	312.6	297.4	349.8	6.3	634.4	937.2	1171.5	175	− 7.2
L39	7.53	20.65	2087	1045	125	81.6	214.2	24	422.2	525.4	156.8	19	− 5.0
L40	7.01	22.83	2939	1474	551.5	130.2	49.6	6	634.4	312.4	1412.6	2	− 9.1
L41	7.17	21.02	2149	1076	191.9	130.8	130.6	5.3	719.8	397.6	315.6	20	− 6.7
L42	7.65	22.05	2440	1221	190.6	145.3	114.4	33.3	898	482.8	174.4	4	− 8.0
L43	7.4	22.9	3220	1612	261.3	167.9	305	6.5	597.8	951.4	327.4	37	− 4.5
L44	7.56	20.96	2230	1116	129.9	38.9	210	2.3	419	444.2	90.8	14	− 6.5
L45	7.56	21.45	2266	1130	123.4	81.2	191.2	2	422.2	482.2	185.5	25	− 7.8
L46	7.22	20.68	4277	2143	246.9	73.9	388	169.4	451.4	1107.6	156.8	170	− 6.0
L47	7.28	20.8	2657	1329	166.7	134.7	185.4	5.2	427	681.6	289.1	45	− 8.9
L48	7.08	23	2804	1404	202	155.7	184.3	5.4	585.6	667.4	374.4	35	− 8.5
L49	7.35	21.7	2203	1104	187.5	113.6	130.8	6.2	512.4	482.8	277.4	42	− 7.4
L50	7.3	19.15	4216	2100	421.6	296.5	179.8	13.1	585.6	667.4	1665.6	8.5	− 8.3
L51	7.27	20.8	4349	2179	195.8	183.7	453.4	7.6	497.8	1300	392.1	65	− 9.3
L52	7.43	20.8	3286	1646	280.6	118	218.8	61.2	402.6	951.4	292.1	70	− 7.8
L53	7.38	20.95	2140	1070	107.8	89.7	192.4	4.4	522.2	468	106.8	28.5	− 7.0
L54	7.18	22.2	2277	1140	184.7	87.5	180.9	12.6	656.4	567.4	100.9	3	− 8.0
L55	7.5	22.7	2148	1076	154.7	123.3	145	3.5	512.4	539.6	186.2	22	− 6.9
L56	7.32	21.5	2650	1327	210.7	122.8	161	4.9	475.8	695.8	233.2	12	− 7.9
L57	7.4	20.8	1873	940	115.4	46.7	185.6	3.2	324.6	439.6	112.6	30	− 7.3
L58	7.4	19.2	4370	2190	314.1	255.1	278.8	85.1	597.8	1178.6	686.2	25	− 6.2
Min	7	14.9	615	308	43.3	31.7	12.4	0.8	244	113	12.6	2	− 9.7
Max	8.4	24	5738	2867	769.5	297.4	671.1	169.4	898	1817.6	1942.1	175	5.2
Mean	7.5	20.5	2453.8	1231.6	210.7	117	175.4	13.8	496.1	584.8	333.7	37.5	− 6.2
Sd	0.3	1.7	1195.6	596.7	128.5	59	133	25.2	116.4	350.1	421.7	39.9	3.3
Campaign 2020													
E1	7.03	20.5	1109	556	110.8	59.7	38.8	3	84.2	429.4	179	10.8	4
E2	7.04	19.6	1030	515	109.8	57.3	34.6	2.5	74.9	378.2	162	16.6	1
E3	7.37	23.14	1537	768	159.4	80.1	63.5	8.5	165.6	390.4	253	58.9	− 1
E4	7.23	21.8	1597	798	160	92.1	67	3.9	159.7	420	303	20.8	− 1
E5	7.12	20.8	1504	753	153.9	94.7	70.9	3.1	163.5	436	274	10.8	− 3
E6	7.85	22	636	319	80.4	19.8	24	1.2	40.4	220	58	30.4	− 2
E7	7.15	21	541	271	102.6	41.6	23.9	2.7	54.1	292.8	119	27.5	− 2
E8	7.6	21	868	434	102.6	41.6	23.2	2.8	50.9	285.5	126	27.4	− 2
E9	7.4	21	806	403	99.2	40.1	22	2.7	50.2	283	125	27.8	− 1
E10	7.4	23	955	480	135.1	62.6	33.5	3.7	67.9	346.5	161	18.8	− 9
E11	7.45	23	3345	1673	243.7	141.3	341.4	10.4	760	320	509.5	79.6	0
E12	7.3	21.5	3930	1966	354.1	89.1	401.3	10	1106.2	380	130	92.7	− 1
E13	7.5	21.6	1353	675	150.5	70	71.5	5.9	183.7	356.2	216	5.4	− 3
E14	7.6	21.75	2008	1002	191.3	85.1	183.2	10.5	432.3	327	245	87.9	− 1
E15	7.2	21.3	2480	1242	235.1	89.6	194.2	24.1	562	680	42	0.9	0
E16	7.2	22	4000	1996	304.1	147.7	394.7	9.8	1150	383.1	246	87.1	1
E17	7.7	23	1754	876	163.1	77.4	140.2	5.1	321.5	322.1	179	49.6	− 5
E18	8	21.5	666	330	89.5	35.3	26.9	2.7	49.4	297.7	53	28.3	− 5
E19	7.8	22.3	2330	1163	189.9	75.9	278.1	13.9	452.2	405	201.3	87.3	− 6
E20	7.6	22	2256	1128	167.6	48.8	263	11.4	678.3	268.4	52	16	2
E21	7.4	22.6	4890	2440	414.4	171.9	470.3	70.7	1502	329.4	252	90.9	− 2
E22	7.5	21.4	1978	920	213	72.2	154.4	6.4	434	488	89	9.1	− 3
E23	8	21.8	1390	695	90.1	48.8	109.2	2.1	255.7	305	68	63.6	5
E24	7.4	22.3	1390	696	114.2	37.2	105	2.2	273.2	278.2	104	33.4	6
E25	7	24	875	438	80.5	41.8	34.6	1.7	73.7	390.4	63	30.8	7
E26	7.3	25	985	493	86.1	47.5	42.2	1.9	99.5	356.2	106	25.9	6
E27	7.3	21.4	2145	1072	196.2	75.3	168.6	10.7	357.4	317.2	361	13.9	− 1
E28	7.5	21	3715	1859	333.9	227.5	236.7	10.4	792.3	490.4	475	19	− 6
E29	7.5	22.8	4580	2290	284	250.8	401	26.6	1305.1	390.4	430	0.4	− 1
E30	7.5	21.3	2321	1164	168.9	100.7	188.5	5.6	481.8	453.8	292	45.4	5
E31	7.4	21.9	3270	1647	263.6	117.8	317.5	3.3	950.1	351.4	137	87.3	0
E32	7.4	22.3	4323	2162	235.7	204.2	431.1	4.9	1300	444.1	61	86.5	− 1
E33	7.1	21	3361	1683	324	212	131	9.3	380.4	405	980	3.1	− 2
E34	7.7	21.2	1198	595	82.8	60.6	61.9	4.1	99.4	350.2	205.2	35.6	6
E35	7.6	21.2	1496	749	103.9	70.1	102.4	16.6	192.9	385.5	32	87.1	− 7
E36	7.9	20.7	2338	1174	277.1	137.9	106.6	4.6	262.5	219.6	720	79.5	− 5
E37	7.3	21.6	4340	2175	355	222	313	3.8	1256	497.8	205	92.3	0
E38	7.9	21	850	424	85.2	14.7	57.3	0.6	95.4	244	49	90.8	7
E39	7.1	22	1482	741	156.8	67.6	57.3	3	178.6	344	251	90.9	4
E40	7.6	19.8	4571	2291	375	285	231	8.5	645.4	348.9	1300	4.5	− 1
E41	7.6	23.6	1148	573	117.9	53.9	34.6	2.2	77.9	297.7	242	34.6	3
E42	7.5	24.5	1440	721	159.4	80	40	2.8	95.3	280.6	403.6	30.9	0
E43	7.4	21.7	3340	1676	676.6	237.8	56.8	9	156.7	273.3	2106	4.5	− 3
E44	7.4	23	2090	1046	252.2	144.9	53	4.4	172.2	348.9	553	15.3	− 9
E45	7.5	26	813	407	68.7	36.2	32.1	2.4	71.4	309.9	67	35.3	7
E46	7.9	21	1603	800	129.5	68.5	108	8.7	256	473.4	95	12.2	0
E47	8.1	22	1013	505	107.1	32.6	53.9	5.1	144.5	236.7	137	47.8	5
E48	7.2	24.3	2897	1450	699.1	125	59.5	5	850	322.1	1231	20.8	7
E49	7.4	22.8	1750	874	142	95.6	115.1	4	222.1	350	380	65.6	2
E50	7.1	20.2	1157	579	101.9	60.1	67	7	142.1	329.4	251.6	0.1	5
E51	7.5	25	1394	697	164.9	71.7	37.4	3.8	84.5	366	246	35.8	− 6
E52	7.3	23.8	2005	1004	143.4	81.4	154	2.8	433.2	390.4	182.8	58.7	6
E53	7.2	22.7	2417	1213	169.5	87	214.4	3.8	571.2	348.9	174	88.3	4
E54	7.4	22	3800	1899	356.9	94	310.1	24.3	1200	209.8	195	90.4	4
E55	7.5	22.3	1970	982	111.7	77.4	191.2	4.6	432.6	480.7	26	60.4	3
E56	7.1	23	2126	1063	123.3	79.6	181.5	8.3	510.8	527	70	1.5	8
E57	7.6	23	2106	1053	159.7	82.2	173	5.4	415.7	331.8	33	62.1	− 9
E58	7.2	24.2	2593	1297	202	112.4	195.2	3.7	534.3	431.9	323	86.1	4
E59	7.3	22.9	2358	1177	178	101.9	208.3	3.4	555.9	309.9	308	88.4	4
E60	7.6	21	865	433	92.3	25.2	26.2	5.8	50.7	200	100	0.7	− 8
E61	8.17	26.7	637	318	85	48.1	35.6	4.1	256	197.5	43.3	3.7	7
Min	7	19.6	541	271	68.7	14.7	22	0.6	40.4	197.5	26	0.1	− 9
Max	8.2	26.7	4890	2440	699.1	285	470.3	70.7	1502	680	2106	92.7	8
Mean	7.5	22.2	2061.1	1029.9	193.2	93.6	143.6	7.5	406.2	354.5	278.4	42.9	0.3
Sd	0.3	1.4	1163.2	582.3	126.6	60.9	120.6	9.8	384.1	88.3	349.5	32.8	4.6

#### Sodium adsorption ratio (SAR)

Another indicator of water quality for agricultural irrigation is the sodium adsorption ratio (SAR). High SAR can cause a deterioration in soil permeability and structure. Furthermore, this can lead to soil salinization in arid climates, which decreases plant capacity through the roots and poor drainage [32–34]. Higher salinity decreases osmotic activity by preventing water from reaching plant branches and leaves, resulting in a lower yield. Groundwater is classified by SAR as low (SAR < 10), medium (10 < SAR < 18), high (18 < SAR < 26), and very high (SAR > 26) sodium hazard. Sodium salinity hazard is due to higher SAR values, which reduce soil water availability, affecting crop growth and reducing the magnesium and calcium nutrient ratio. [34]. Salinity hazards are classified as very high salinity water (C4), high salinity water (C3), medium salinity water (C2), and low salinity water (C1). However, alkalinity hazards are divided into four categories: very high sodium water (S4), high sodium water (S3), medium sodium water (S2), and low sodium water (S1). However, SAR was employed as an alkalinity hazard, while EC was used as a salinity hazard [29].

**Fig. 4:**
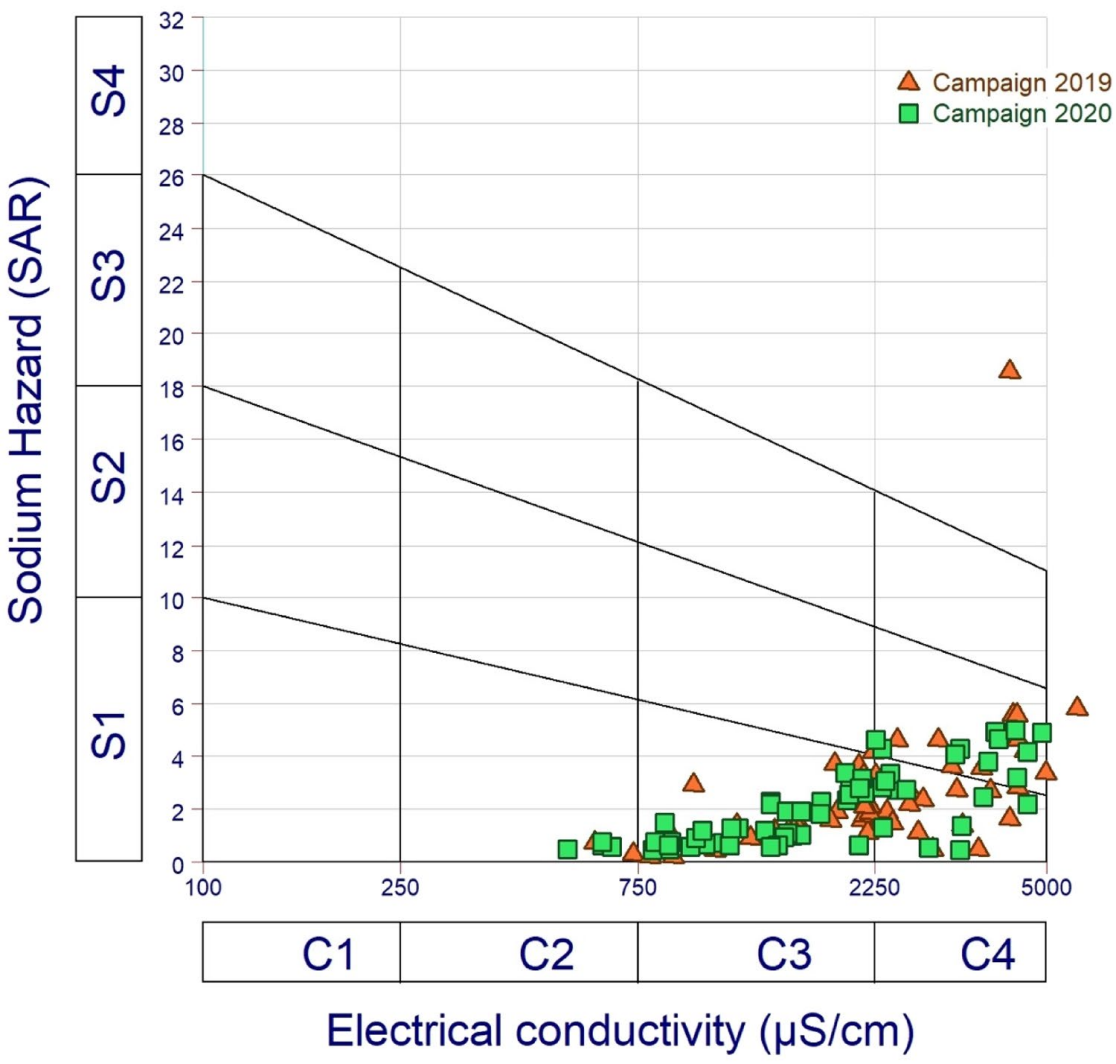
Irrigation water classification diagram United States Salinity Laboratory (USSL)

Groundwater samples from the 2019 campaign revealed SAR values ranging from 0.22 to 18.53, with an average of 2.57. (Table 3). SAR values below 10 are found in all of the examined samples, which are mostly found at the foot of the surrounding hills, indicating excellent irrigation water quality and, as a result, no threat (Fig. 4). One sample (E4) obtained from wells near Meskala village in the center has SAR values above 10, indicating precipitation produced by salt leaching and dissolving, which might degrade soil texture and impair plant survival and growth [29]. Groundwater samples were divided into three categories based on the plot of SAR versus electrical conductivity (US salinity diagram) [39]. (Fig. 4). The C2–S1 classes are represented by 3.44% of samples in this plot, highlighting high salinity and low to medium SAR. However, C4–S1 and C4–S2 samples account for 41.37% of all samples, indicating high salinity and medium sodium hazard. Furthermore, 55.17% of water samples were classified as C3–S1, indicating significant salinity and sodium toxicity.

**Table 3:** Values of SAR. Na%. and WQI in the Meskala-Ouazzi sub-basin for the groundwater of the campaign 2019 and 2020

Campaign 2019							Campaign 2020						
Sample	SAR	(Na %)	WQI	Classification	IWQI	Classification	sample	SAR	(Na %)	WQI	Classification	IWQI	Classification
L1	4.19	33.13	295.68	Very poor water	143.32	poor water	E1	0.74	14.46	90.7	Good water	51.02	Good water
L2	2.81	32.79	147.86	Poor water	73.53	Good water	E2	0.67	13.34	85.65	Good water	66.66	Good water
L3	3.08	35.2	159.57	Poor water	83.64	Good water	E3	1.02	17	126.32	poor water	65.78	Good water
L4	18.54	84.75	199.29	Poor water	175.72	poor water	E4	1.04	16.23	118.04	poor water	64.00	Good water
L5	1.68	20.42	167.11	Poor water	71.51	Good water	E5	1.11	16.98	112.3	poor water	50.27	Good water
L6	0.73	15.71	62.23	Good water	46.86	Excellent water	E6	0.62	16	60.91	Good water	42.33	Excellent water
L7	1.93	24.37	157.36	Poor water	73.19	Good water	E7	0.5	11.49	69.29	Good water	46.86	Excellent water
L8	2.7	26.83	226.39	Very poor water	96.70	Good water	E8	0.49	11.23	69.4	Good water	47.54	Excellent water
L9	2.16	25.07	146.14	Poor water	71.58	Good water	E9	0.47	11.06	73.92	Good water	53.40	Good water
L10	0.45	7.9	99.78	Good water	39.76	Excellent water	E10	0.6	11.54	85.65	Good water	97.23	Good water
L11	2.94	39.27	123.13	Poor water	42.80	Excellent water	E11	4.31	38.86	214.16	Very poor water	100.75	poor water
L12	1.93	26.03	129.1	Poor water	62.84	Good water	E12	4.94	41.47	235.31	Very poor water	66.36	Good water
L13	4.62	38.05	279.82	very poor water	144.35	poor water	E13	1.21	19.73	100.11	Good water	81.18	Good water
L14	4.62	44.98	157.46	Poor water	98.29	Good water	E14	2.77	33.24	156.33	Poor water	93.06	Good water
L15	4.63	45.44	217.26	Very poor water	98.35	Good water	E15	2.73	32.18	168.25	Poor water	102.26	Poor water
L16	0.33	6.34	76.36	Good water	37.10	Excellent water	E16	4.64	38.93	240.92	Very poor water	77.89	Good water
L17	0.23	3.8	104.2	Poor water	40.41	Excellent water	E17	2.26	30.04	125.82	Poor water	59.82	Good water
L18	0.22	4.36	86.48	Good water	38.50	Excellent water	E18	0.61	14.39	68.94	Good water	95.49	Good water
L19	1.21	17.11	123.63	Poor water	56.09	Good water	E19	4.31	44.2	170.65	Poor water	83.47	Good water
L20	1.34	19.86	113.64	poor water	57.56	Good water	E20	4.6	48.66	132.65	Poor water	112.23	Poor water
L21	1.62	18.59	167.58	Poor water	81.93	Good water	E21	4.9	39	309.2	Unfit for drinking	82.66	Good water
L22	3.58	32.85	236.84	Very poor water	118.39	Poor water	E22	2.33	29.34	131.57	Poor water	80.72	Good water
L23	5.83	41.71	322.27	Unfit for drinking	178.33	Poor water	E23	2.3	36.08	105.52	Poor water	64.49	Good water
L24	0.78	13.44	99.43	Good water	45.41	Excellent water	E24	2.18	34.55	96.58	Good water	52.09	Good water
L25	1.05	16.06	117.82	Poor water	54.27	Good water	E25	0.78	17.2	79.9	Good water	56.82	Good water
L26	1.17	13.51	203.65	Very poor water	87.36	Good water	E26	0.91	18.68	82.36	Good water	71.74	Good water
L27	1.19	17.76	118.43	Poor water	48.42	Excellent water	E27	2.59	32.24	136.67	Poor water	102.82	Poor water
L28	1.5	20.39	130.01	Poor water	53.38	Good water	E28	2.45	22.99	222.75	Very poor water	112.27	Poor water
L29	1.12	13.73	173.24	Poor water	57.75	Good water	E29	4.18	34.24	252.01	Very poor water	92.08	Good water
L30	1.6	22.09	152.58	Poor water	70.11	Good water	E30	2.84	33.3	158.85	Poor water	94.61	Good water
L31	1.41	21.73	112.94	Poor water	42.43	Excellent water	E31	4.09	37.82	203.19	Very poor water	114.46	Poor water
L32	1.22	14.85	145.45	Poor water	58.45	Good water	E32	4.96	39.79	245.18	Very poor water	88.12	Good water
L33	0.5	4.85	294.53	Very poor water	85.75	Good water	E33	1.39	15.01	207.95	Very poor water	70.36	Good water
L34	1.43	13.39	277.11	very poor water	108.58	poor water	E34	1.26	23.48	96.24	Good water	75.89	Good water
L35	1.4	21.87	99.15	Good water	47.45	Excellent water	E35	1.9	30.82	125.47	Poor water	80.02	Good water
L36	0.91	14.5	108.63	Poor water	42.44	Excellent water	E36	1.31	15.89	173.45	Poor water	109.48	Poor water
L37	1.31	25.87	74.12	Good water	36.44	Excellent water	E37	3.21	27.59	262.45	Very poor water	64.78	Good water
L38	3.4	27.44	347.13	Unfit for drinking	135.99	Poor water	E38	1.51	31.47	88.21	Good water	60.01	Good water
L39	3.66	40.71	144.02	Poor water	73.15	Good water	E39	0.96	16.1	128.38	poor water	109.41	poor water
L40	0.49	5.32	241.87	Very poor water	70.32	Good water	E40	2.19	19.58	263	Very poor water	59.21	Good water
L41	1.78	21.71	165.82	Poor water	60.52	Good water	E41	0.66	13.14	92.69	Good water	59.08	Good water
L42	1.52	18.24	189.83	Poor water	75.06	Good water	E42	0.65	11.08	108.98	Poor water	91.12	Good water
L43	3.62	32.93	216.2	Very poor water	109.15	poor water	E43	0.48	4.82	268.95	Very poor water	67.62	Good water
L44	4.15	48.4	126.71	Poor water	69.45	Good water	E44	0.66	8.98	142.85	Poor water	55.69	Good water
L45	3.28	39.21	137.89	Poor water	71.87	Good water	E45	0.78	18.54	74.18	Good water	83.92	Good water
L46	5.56	42.61	339.46	Unfit for drinking	128.13	Poor water	E46	1.91	28.91	113.7	Poor water	66.29	Good water
L47	2.59	29.22	171.2	Poor water	82.52	Good water	E47	1.17	23.57	87.92	Good water	82.19	Good water
L48	2.37	25.83	189.36	Poor water	81.12	Good water	E48	0.54	5.67	245.79	Very poor water	73.92	Good water
L49	1.86	23.17	159.57	Poor water	67.60	Good water	E49	1.83	25.47	136.38	Poor water	56.81	Good water
L50	1.64	14.6	295.91	Very poor water	111.67	Poor water	E50	1.3	23.57	88.53	Good water	61.43	Good water
L51	5.59	44.02	255.45	Very poor water	140.96	Poor water	E51	0.61	10.88	108.49	Poor water	78.33	Good water
L52	2.76	27.35	234.68	Very poor water	107.14	Poor water	E52	2.55	32.83	140.04	Poor water	82.20	Good water
L53	3.31	39.41	139.93	poor water	67.32	Good water	E53	3.34	37.63	163.29	Poor water	89.70	Good water
L54	2.75	31.99	156.28	Poor water	69.27	Good water	E54	3.77	35.58	230.85	Very poor water	92.02	Good water
L55	2.11	26	1145.64	Unfit for drinking	71.79	Good water	E55	3.4	41.39	138.59	Poor water	85.74	Good water
L56	2.18	25.25	163.95	Poor water	81.18	Good water	E56	3.13	38.96	132.97	Poor water	79.49	Good water
L57	3.69	45.48	117.83	Poor water	63.23	Good water	E57	2.77	34.22	134.89	Poor water	87.62	Good water
L58	2.83	23.8	304.8	Unfit for drinking	134.15	Poor water	E58	2.73	30.76	180.74	Poor water	82.30	Good water
							E59	3.08	34.63	166.1	Poor water	46.40	Excellent water
							E60	0.62	16.17	59.92	Good water	60.61	Good water
							E61	0.76	16.78	61.64	Good water	61.18	Good water

SAR values for the Meskala-Ouazzi sub-basin groundwater samples range from 0.47 to 5, with an average value of 2 for the 2020 campaign. (Table 3). SAR values below 10 are found in all of the examined samples, which are mostly found at the foot of the surrounding hills, indicating excellent irrigation water quality and, as a result, no threat (Fig. 4). Groundwater samples were divided into three categories based on the plot of SAR versus electrical conductivity (US salinity diagram) [39]. (Fig. 4). The C2–S1 classes are represented by 6.55% of samples in this plot, highlighting low sodium water (S1 to medium SAR). However, 36.05% of samples are classified as C4–S1 or C4–S2, indicating low salinity and a medium sodium threat. Furthermore, 57.40% of water samples were classified as C3–S1, indicating significant salinity and sodium toxicity. To summarize, samples that fall into the C4–S1, C4–S2, and C4–S3 categories are undesirable for irrigation in all soil types, except for extremely permeable soil with considerable drainage capacity and the selection of salt-tolerant plants [33]. In reality, using these water types for irrigation will decrease crop output and cause soil structure and texture deterioration. On the other hand, groundwater samples in the C3–S1 group should only be used to irrigate salt-tolerant crops produced on well-drained soils with high permeability under regular salinity monitoring [33]. Thus, 95% are in the C3 and C4 classes of the 2020 campaign, which have the highest salinity and medium to high sodium risks and can only be used on plants that tolerate high salinity.

**Fig. 5:**
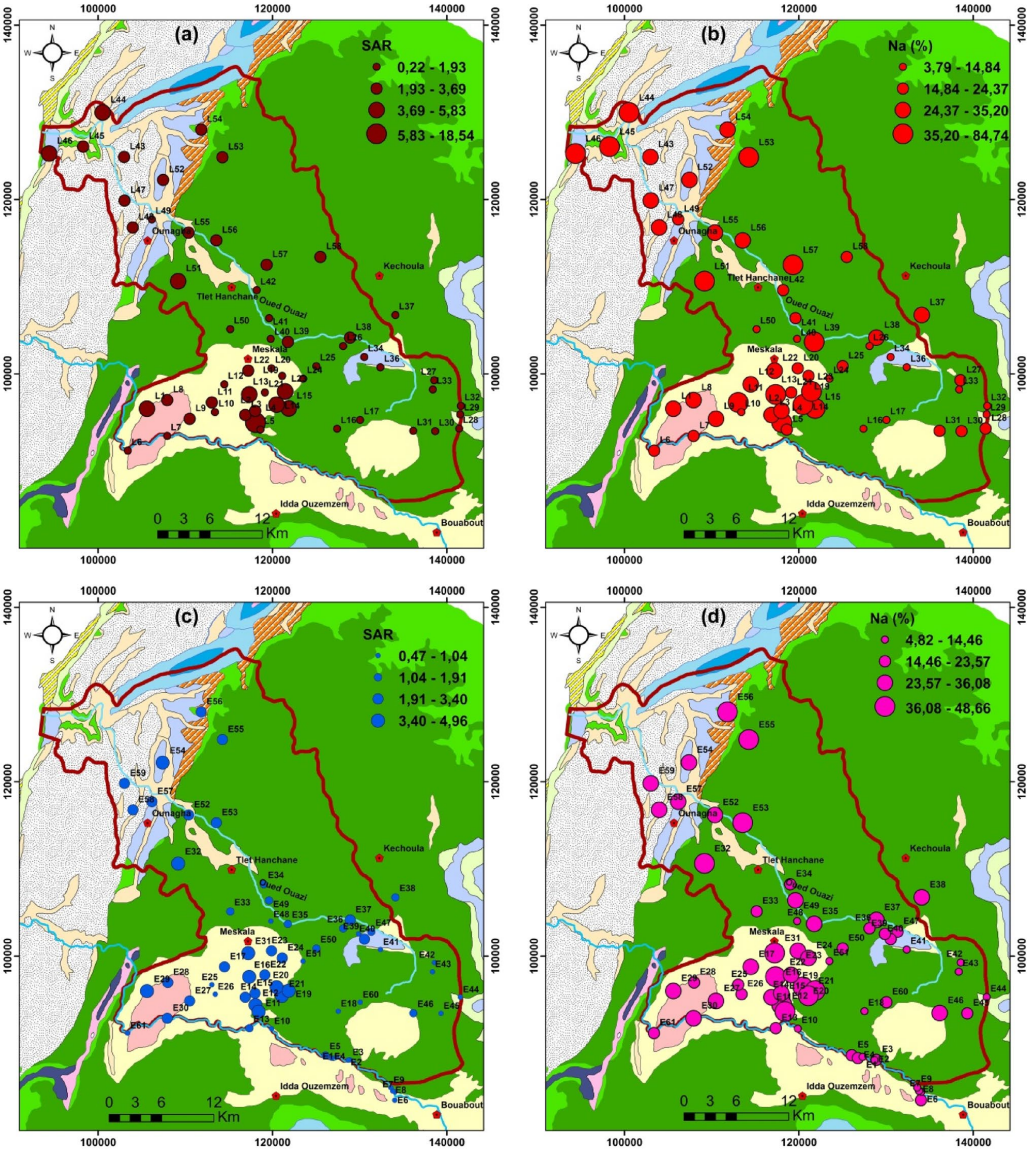
Spatial distribution map of SAR and Na (%) of the campaign 2019 and 2020, respectively

The distribution map of SAR for the campaigns 2019 and 2020 (Figs. 5a, 5c) indicates that most of the groundwater samples cannot be utilized for safe drinking due to evaporation, rock–water interaction, and reverse ion exchange processes in the north-western parts as well as saltwater intrusion in downstream of the study area toward the Atlantic Ocean.

#### Sodium percentage (%Na)

Excess salt can limit soil permeability; hence the sodium percentage was computed to offer information about the suitability of groundwater for irrigation [29]. Large levels of sodium in groundwater (over 60%) can generate sodium accumulations, disrupting soil formation [12, 33, 35, 36].

**Fig. 6:**
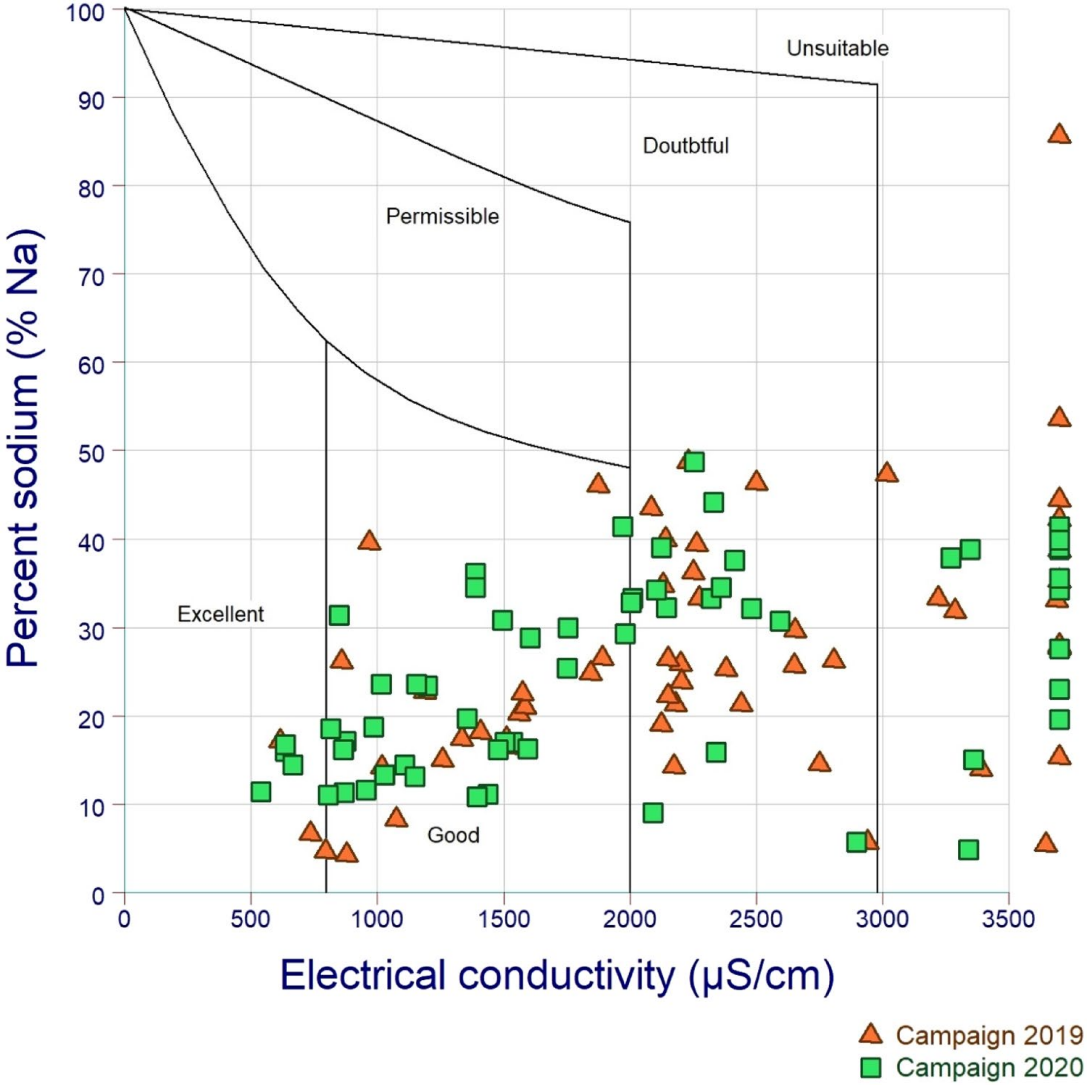
Suitability of groundwater for irrigation in the Wilcox diagram

For the 2019 campaign, the %Na in groundwater samples from the Meskala-Ouazzi sub-basin ranges from 4.30 to 85.60%, with a mean value of 27.18%. The groundwater samples of the study area have been divided into four categories based on the Wilcox diagram, which displays the evolution of sodium percent of EC. About 29.31% of groundwater samples collected from wells in the recharge area's upstream section fell into the "Good to Permissible" category, indicating minimal mineralization (Fig. 6). In addition, 37.93% of samples fell into the "Doubtful to Permissible" group, indicating modest mineralization. Almost half of the groundwater samples (27.58%) are unfit for irrigation, indicating significant mineralization (EC > 3000 S/cm). Only 3.44% of groundwater samples are deemed ideal for irrigation (Fig. 6).

For the 2020 campaign, the %Na in groundwater samples from the Meskala-Ouazzi sub-basin ranges from 4.82 to 48.66%, with a mean value of 25.1%. The groundwater samples of the Meskala-Ouazzi sub-basin have been divided into four categories based on the Wilcox diagram, which displays the evolution of sodium percent EC. A total of 47.54% of groundwater samples obtained from wells in the recharge area's downstream section fell into the "Good to Permissible" category, indicating little mineralization (Fig. 6). In addition, 24.59% of samples fell into the "Doubtful to Permissible" group, indicating modest mineralization. Almost half of the groundwater samples (21.31%) are unfit for irrigation, indicating excessive mineralization (EC > 3000 S/cm). Only 4.91% of groundwater samples are deemed suitable for irrigation (Fig. 6). The Wilcox diagram observed that 6.5% of the samples from the 2020 campaign are excellent to use for irrigation, 53.25% of the samples lie in the acceptable class, about 20.11% of the samples lie in the doubtful region, and about 20.14% of the samples lie in the unsuitable region, meaning they cannot be used for irrigation due to the adverse effects they may cause.

According to this classification, most groundwater collected in the Meskala-Ouazzi sub-basin of the campaigns 2019 and 2020 (Fig. 5b, 5d) is suitable for irrigation.

#### Irrigation water quality index

**Fig. 7:**
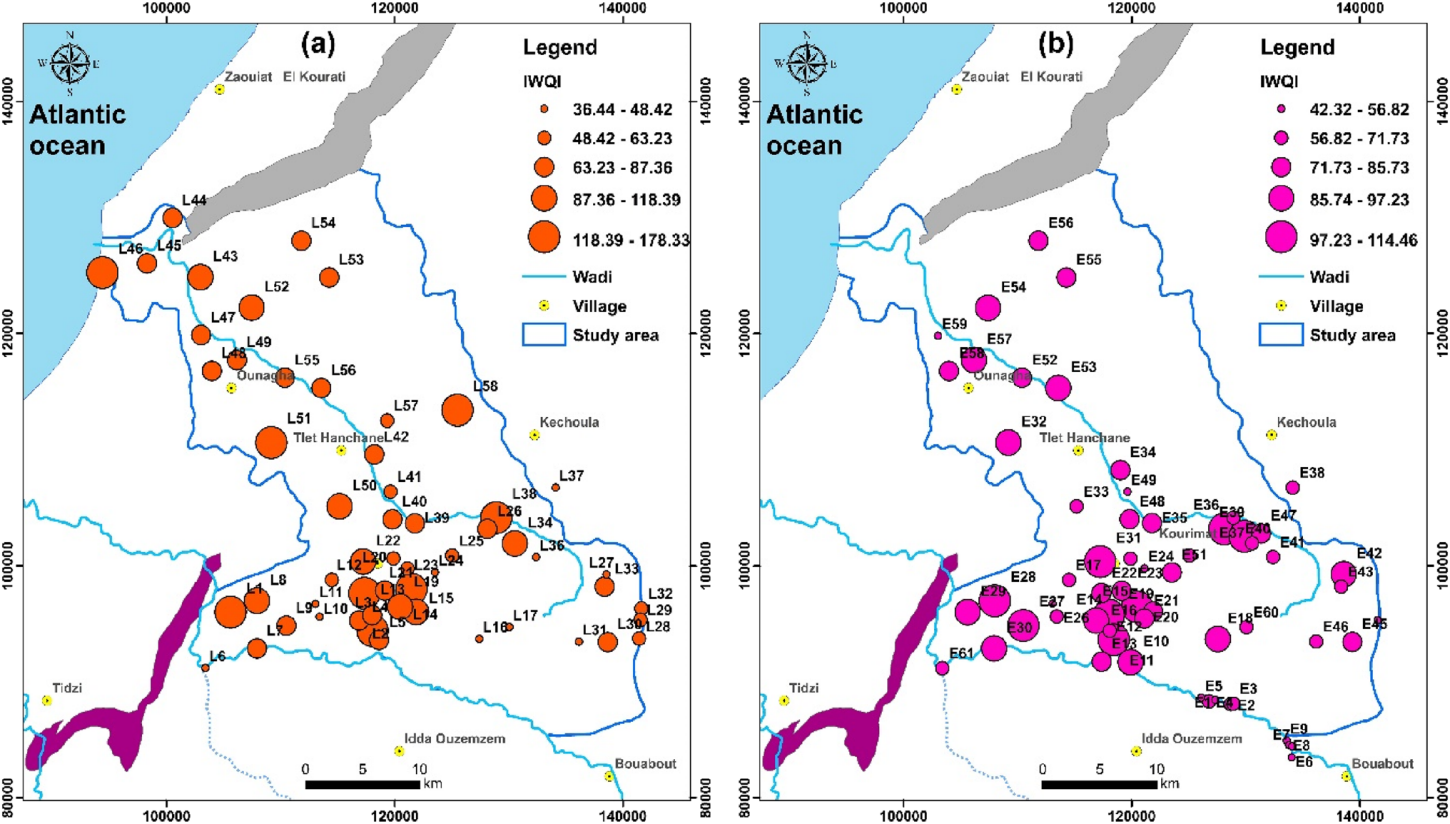
Spatial distribution map of IWQI values in the Meskala-Ouazzi sub-basin in the campaigns 2019 (**a**) and 2020 (**b**)

Irrigation water quality index is a technique for determining the suitability of plant life and soil constituents [12, 36]. It has also been shown that the composite impacts the mineral constituents' effects in groundwater monitoring. The worldwide standard is used to determine irrigation appropriateness for groundwater quality. The types of soil influence a plant's growth and the quality of the water it consumes, with water being the most important factor. When poor quality water is used for farming, it impacts crop output. Intense agricultural methods, as well as a higher rate of chemical fertilizers that use saline on the coast and mix with groundwater, have significantly impacted groundwater quality. The IWQI model was presented in this study based on the integration of eleven hydrogeological groundwater quality parameters: pH, Cl, NO_3_, HCO_3_, %Na, sodium adsorption ratio (SAR), permeability index (PI), magnesium hazard (MH), kelly index (KI), potential salinity (PS), and electrical conductivity (EC). This irrigation groundwater quality criterion was derived based on recommendations published by [36], and water quality is categorized into five categories: excellent (IWQI < 50), good (50 < IWQI < 100), poor (100 < IWQI < 200), extremely poor (200 < IWQI < 300), and improper (IWQI > 300) [36]. According to the results, the IWQI values for the two campaigns (2019 and 2020) varied from 40.88 to 154.61, with an average of 76.56. When compared to water quality classification, it was discovered for the 2019 campaign that groundwater samples were mostly "excellent water" (6.2%), "good quality water" (75.6%), and "bad water" (7.2%) (Table 3) (Fig. 7a). Groundwater samples for the 2020 campaign were mostly "excellent water" (8.2%), "good quality water" (83.6%), and "poor water" (8.2%) (Table 3) (Fig. 7b).

**Fig. 8:**
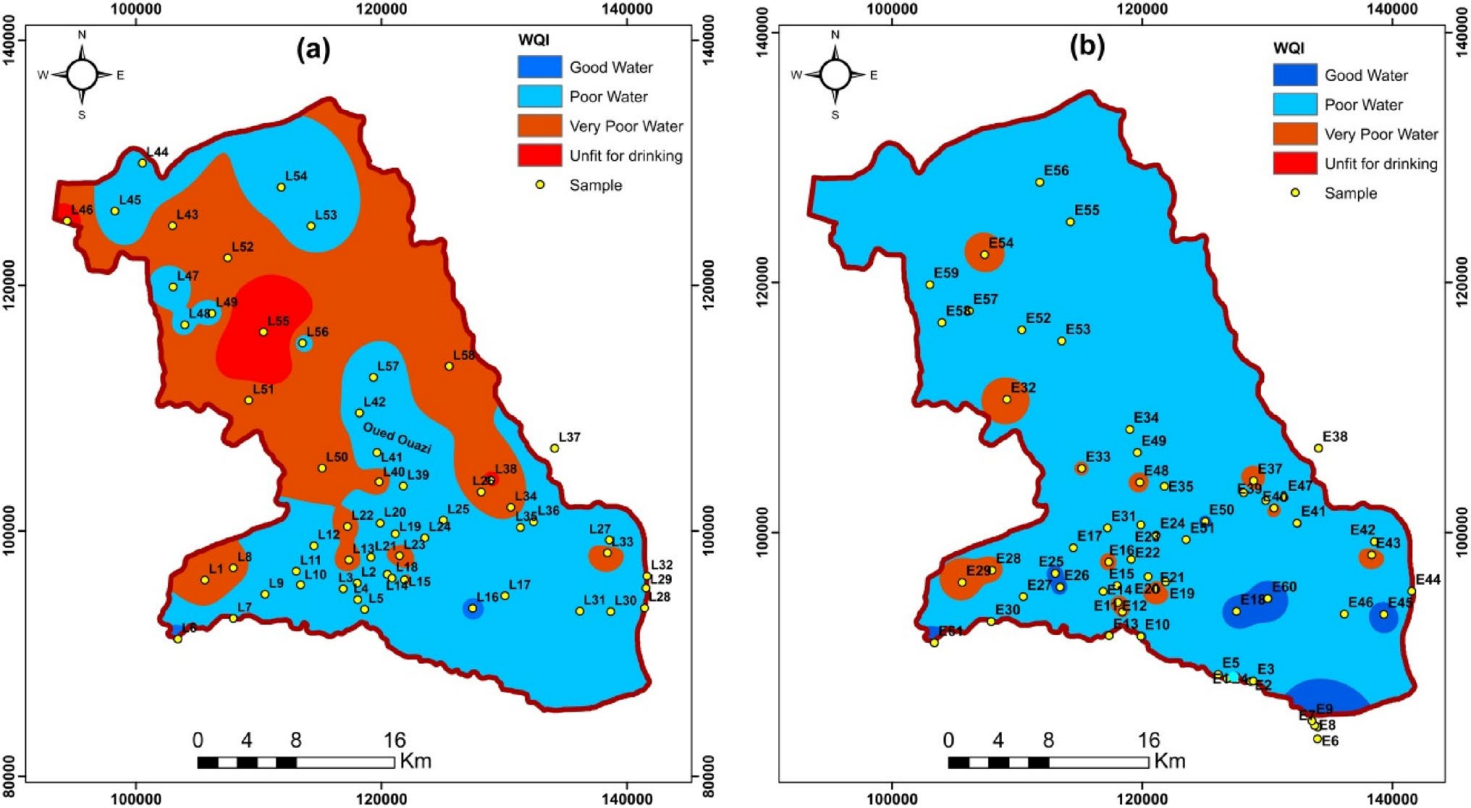
Spatial distribution map of WQI values in the Meskala-Ouazzi sub-basin in the campaigns 2019 and 2020

To assess the geographic variance of groundwater quality, using ArcGIS 10.2 and interpolation techniques (IDW). To construct the different thematic spatial maps, Fig. 8 shows the spatial distribution of IWQI for the three campaigns in 2019 and 2020. These maps show where areas of poor groundwater quality are located so that treatment procedures can be implemented to improve quality by increasing crop productivity.

### Quality of drinking water

The WQI was calculated using the values of eleven physicochemical parameters: pH, EC, TDS, Ca, Mg, Na, K, Cl, HCO_3_, SO_4_, and NO_3_ as only these eleven parameters were considered [13, 14], which are very important for the quality of drinking water. The variation of WQI at different sites in the pre-COVID-19 during and after the lockage periods is shown in Figs. 8a and 8b for the 2019 and 2020 campaigns, respectively.

According to the WQI calculations for the 2019 campaign, 12.07% of the sample sites have good water. On the other hand, more than 56.9% of the sample is poor water, 22.41% of the sample sites have very poor water, and 8.62% are unfit for drinking (Table 3) (Fig. 8a). It can be observed from this graph (Fig. 8a) that there is no excellent water in the study area.

According to WQI for the 2020 campaign during lockdown COVID-19, the total study area has been split into 32.8% (good areas), 44.3% (poor areas), 21.3% (very poor areas), and 1.6% (unfit areas), respectively, and no excellent groundwater areas have been identified (Table 3) (Fig. 8b). Additionally, Fig. 8b showed that water quality has improved compared to the old 2019 campaign in some places.

The spatial distribution of WQI in the Meskala-Ouazzi sub-basin of groundwater samples collected in 2019 and 2020 is represented in Fig. 8. It is evident that in most areas of the sub-basin of Essaouira, the phreatic water was of poor or good quality. Following the results of the two years of water quality index, we observed that water quality is deteriorating, particularly in the downstream part of the Essaouira basin.

The confinement due to COVID-19 has significantly improved the water quality of the Meskala-Ouazzi sub-basin. Instead, approximately 50% of sites showed improved water quality when assessed through the calculation of the Water Quality Index. It is obvious from the spatial distribution maps (Fig. 8a, 8b) that the groundwater in the study area is not of acceptable quality for human consumption.

In the Meskala-Ouazzi sub-basin, areas were determined to present “poor”, “very poor”, and “unfit” quality for consumption should not be used for drinking without some prior treatment; otherwise, there could be various health problems. Water quality is poor due to several factors, such as a combination of natural (interaction of water with the geological environment) and anthropogenic (infiltration of agrochemicals, sewage and overexploitation of wells that produce seawater intrusion) processes.

### Stable isotopes (δ^18^O, δ.^2^H) 

**Fig. 9:**
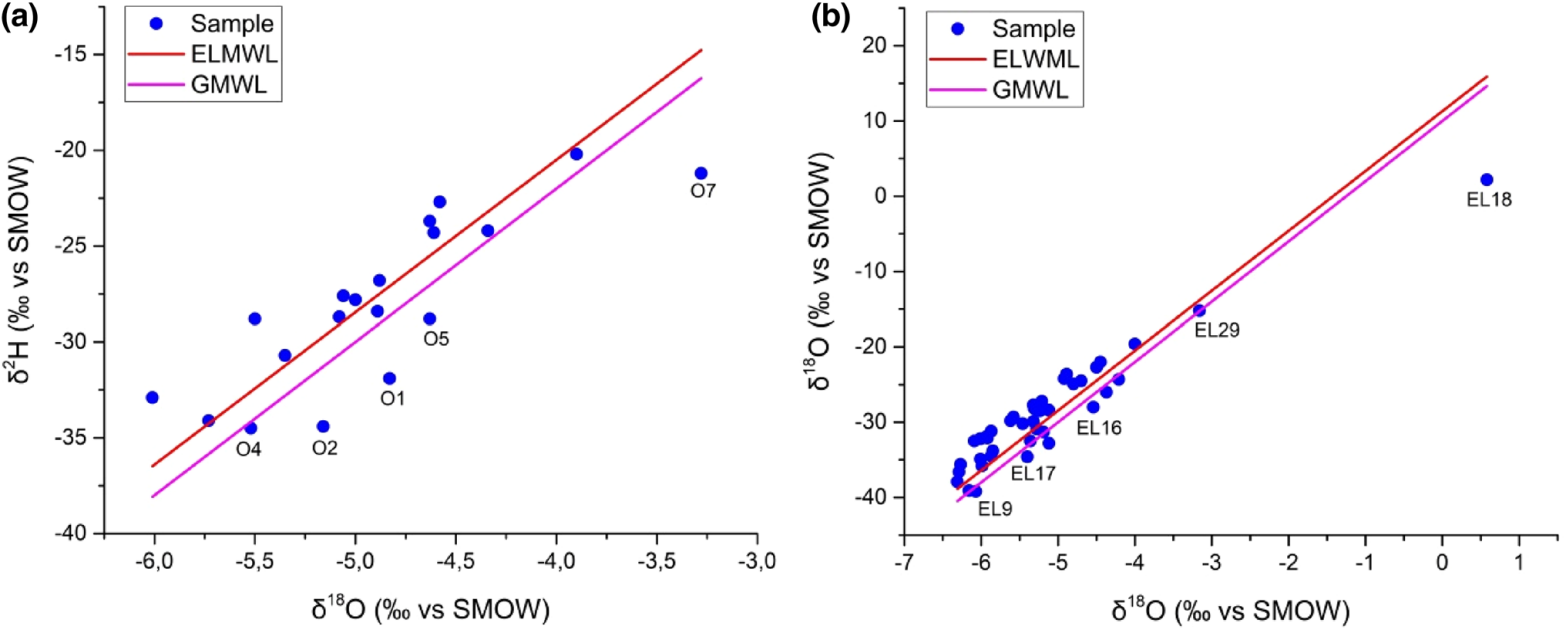
Plot of δ^2^H vs δ^18^O for the groundwater for the campaign 2016a and the campaign 2020b

Water molecules have stable isotopes of oxygen δ^18^O and hydrogen δ^2^H, which include origin characteristics and, therefore, can be used to trace the source and mode of transport of water molecules [37–40]. These isotopes can also be used to identify precipitation's recharge mechanisms [40]. Several locations (spring, borehole, dam and well) of the study area were collected and studied during the 2016 and July of 2020 campaigns to assess the source of aquifer recharge. The results of the isotope analyses are shown in Table 4. The stable isotope ratios of the water molecules of campaign 2016 range between − 6.01 and − 3.28 for oxygen-18 levels, with an average of − 4.89, and between − 34.5 and 20.2 for deuterium contents, with an average of − 27.98 (Fig. 9a) (Table 4). The stable isotope ratios of the water molecule range between − 6.31 and 0.58 for oxygen-18 levels, with an average of − 5.18, and between − 39.2 and 2.2 for deuterium contents, with an average of − 29.04 for the campaign 2020 (Table 4) (Fig. 9b).

**Table 4:** Statistical summary of the stable isotopic values of the campaign 2016 and 2020

Sample	pH	T	EC	NO_3_	Cl	δ^2^H	δ^18^O
		°C	µS/cm			‰ vs. SMOW	
Campaign 2016							
O1	7.2	19.5	2190	1.27	7.98	− 31.9	− 4.83
O2	7.8	21	1188	0.52	2.17	− 34.4	− 5.16
O3	7.4	19.2	1300	0.47	3.35	− 34.1	− 5.73
O4	7.3	22.1	1249	0.69	3.52	− 34.5	− 5.52
O5	7.5	19.8	803	1.3	2.35	− 28.8	− 4.63
O6	7.7	18.5	2050	2.05	5.79	− 28.4	− 4.89
O7	7.5	18	3250	1.55	22.56	− 21.2	− 3.28
O8	6.9	22.1	3050	0	3.52	− 32.9	− 6.01
O9	7.1	23.7	1800	0.32	8.02	− 28.8	− 5.5
O10	7.5	20.9	1883	1.58	10.25	− 27.6	− 5.06
O11	7.3	23	4150	1.83	29.27	− 24.3	− 4.61
O12	7.1	23.4	2550	0	8.27	− 30.7	− 5.35
O13	7.4	23.3	1894	1.07	7.73	− 24.2	− 4.34
O14	7.4	21.2	1933	0.58	8.01	− 26.8	− 4.88
O15	7.2	22.1	2020	0	11.3	− 22.7	− 4.58
O16	7.2	24	2550	0.97	12.66	− 28.7	− 5.08
O17	7.1	23.2	3100	0.99	17.57	− 27.8	− 5
O18	7.3	20.8	3150	1.13	24.28	− 23.7	− 4.63
O19	7.4	20.5	4450	6.31	29.08	− 20.2	− 3.9
Min	6.9	18	803	0	2.17	− 34.5	− 6.01
Max	7.8	24	4450	6.31	29.27	− 20.2	− 3.28
Mean	7.33	21.38	2345.26	1.19	11.46	− 27.98	− 4.89
Campaign 2020							
EL1	7.03	20.5	1109	0.2	2.4	− 34.5	− 5.87
EL2	7.04	19.6	1030	0.3	2.1	− 34.9	− 6.01
EL3	7.37	23.14	1537	0.9	4.7	− 33.8	− 5.85
EL4	7?23	21.8	1597	0.3	4.5	− 31.3	− 5.19
EL5	7.12	20.8	1504	0.2	4.6	− 32.5	− 5.36
EL6	7.85	22	636	0.5	1.1	− 32.8	− 5.12
EL7	7.15	21	541	0.4	1.5	− 37.9	− 6.31
EL8	7.6	21	8.68	0.4	1.4	− 39.1	− 6.16
EL9	7.4	21	806	0.4	1.4	− 39.2	− 6.07
EL10	7.4	23	955	0.3	1.9	− 31.9	− 5.93
EL11	7.45	23	3345	1.3	21.4	− 29.9	− 5.32
EL12	7.5	21.6	1353	0.1	5.2	− 30.9	− 5.28
EL13	7.7	23	1754	0.8	9.1	− 26	− 4.37
EL14	7.6	21	865	0.0	1.4	− 32.2	− 6.01
EL15	8	21.5	666	0.5	1.4	− 31.2	− 5.87
EL16	8	21.8	1390	1.0	7.2	− 28	− 4.54
EL17	7.3	25	985	0.4	2.8	− 34.6	− 5.4
EL18	8.17	26.7	637	0.1	7.2	2.2	0.58
EL19	7.4	21.9	3270	1.4	26.8	− 24.3	− 4.21
EL20	7.4	22.3	4323	1.4	36.7	− 24.9	− 4.8
EL21	7.7	21.2	1198	1.4	5.4	− 29.3	− 5.58
EL22	7.6	21.2	1496	1.3	7.4	− 32.5	− 6.09
EL23	7.9	20.7	2338	1.5	2.7	− 30.2	− 5.46
EL24	7.9	21	850	0.6	2.2	− 27.2	− 5.21
EL25	7.6	23.6	1148	0.5	2.7	− 32.1	− 5.92
EL26	7.5	24.5	1440	0.2	4.9	− 35.8	− 5.99
EL27	7.4	23	2090	1.1	6.3	− 35.6	− 6.27
EL28	7.4	22.8	1750	0.0	4.0	− 36.6	− 6.29
EL29	7.1	20.2	1157	0.6	2.4	− 15.2	− 3.16
EL30	7.5	25	1394	0.9	12.2	− 29.8	− 5.62
EL31	7.3	23.8	2005	1.4	16.1	− 28.2	− 5.31
EL32	7.2	22.7	2417	1.5	33.9	− 28.4	− 5.12
EL33	7.4	22	3800	1.0	6.3	− 22.7	− 4.5
EL34	7.5	22.3	1970	0.0	14.4	− 24.2	− 4.92
EL35	7.1	23	2126	1.0	11.7	− 22	− 4.45
EL36	7.6	23	2106	0.0	10.7	− 24.5	− 4.7
EL37	7.3	23	2993	*	*	− 28.5	− 5.24
EL38	7.5	22.3	1891	*	*	− 23.6	− 4.89
EL39	7.2	21.5	5285	*	*	− 19.6	− 4
EL40	7.7	21	1752	*	*	− 27.7	− 5.32
Min	7.03	19.6	8.68	*	*	− 39.2	− 6.31
Max	8.17	26.7	5285	*	*	2.2	0.58
Mean	7.48	22.24	1737.94	*	*	− 29.04	− 5.18

*No measurement

The Local Meteoric Water Line (LMWL) (δ^2^H = 7.55 × δ^18^O + 9.2 [41]) and the Global Meteoric Water Line (GMWL) (δ^2^H = 8 × δ^18^O + 10 [37] have been located on the correlation diagram of oxygen-18 versus deuterium (Fig. 9). The majority of the water samples were close to both the local meteoric water line and the global meteoric water, showing that both the groundwater and atmospheric precipitation were the primary sources.

This recharge is visible throughout the upstream sector of the study area, particularly in the Bouabout region and in the Meskala region to the southwest. Furthermore, the apparent low salinities of the samples tend to support this conclusion. Other samples with more stable isotopes, such as the dam water and a few samples in the downstream half of the study area, fall below the GMWL. Groundwater samples in the discharge area to the west and south of the research area showed higher δ^2^H and δ^18^O, indicating that surface water sources are impacting groundwater. This highlights the role of evaporation and other processes in groundwater mineralization.

#### Isotopic evidence of evaporation

**Fig. 10:**
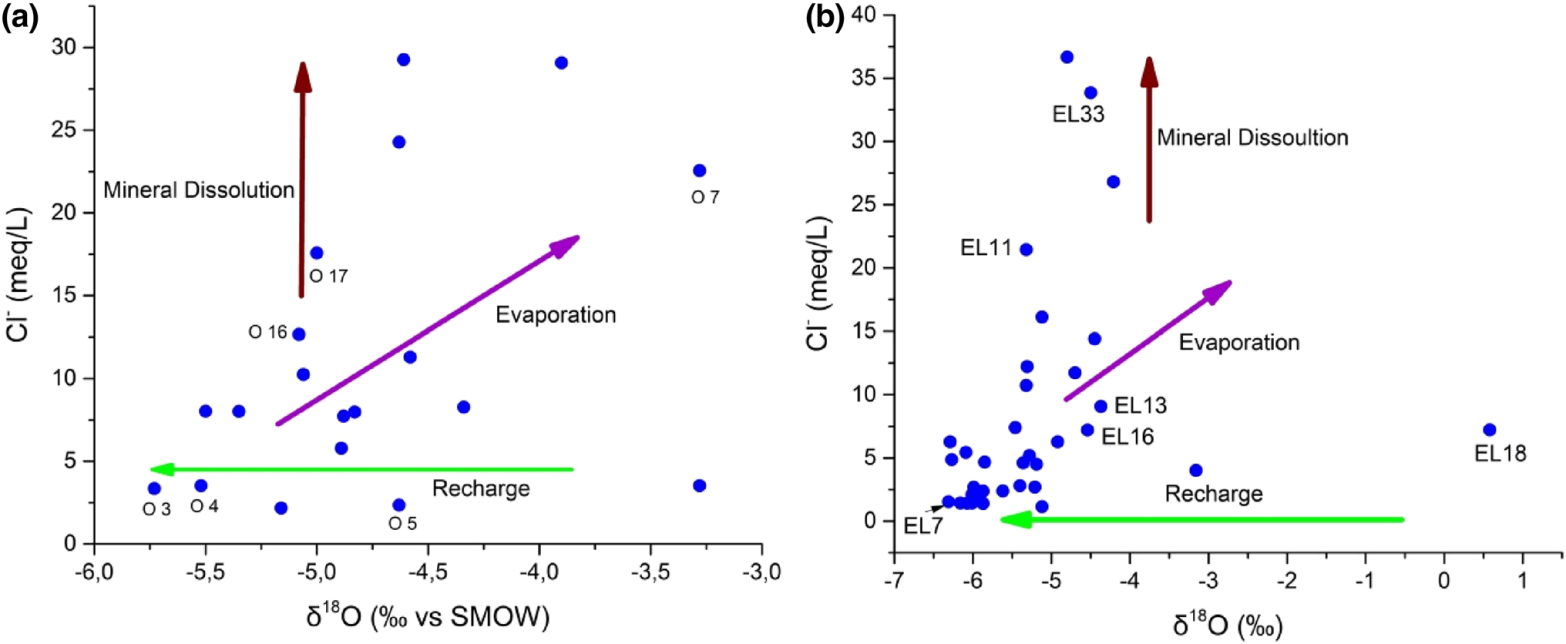
Plot of Cl^−^ vs δ.^18^O for the groundwater for the campaign 2016 (**a**) and the campaign 2020 (**b**)

Chloride concentrations were plotted against the groundwater's δ^18^O signature to better understand the recharging mechanism and the impact of evaporation on isotopic signatures (Fig. 10a,b) for the two campaigns 2016 and 2020. When it comes to determining the mechanism of groundwater recharge, such connections are extremely useful. Any rise in salt concentrations owing to evaporation should be shown in a linear relationship with an enrichment in δ^18^O signatures [42]. The dissolution of salts, on the other hand, results in an increase in salinity but not in the fractionation of water molecules. The following are the key conclusions that may be drawn from δ^18^O–Cl plots:

Chloride concentrations were plotted against the δ^18^O signature of the groundwater to understand the recharge mechanism and the impact of evaporation on isotopic signatures (Fig. 10a, b) for both the 2016 and 2020 campaigns. When it comes to determining the mechanism of groundwater recharge, these connections are extremely useful. Any increase in salt concentrations due to evaporation should show a linear relationship with the enrichment of δ^18^O signatures [42]. On the other hand, the dissolution of salts increases salinity but not in the fractionation of water molecules.

The link between stable chloride and water isotopes (δ^18^O, δ^2^H) is employed to provide a reliable interest by combining chemical and isotopic data to confirm the fundamental processes governing groundwater salinity. Many previous research studies [15–17, 42] in arid and semi-arid areas have found that groundwater salinity is mainly determined by dissolution or evaporation. Indeed, the graph shows a significant increase in Cl^−^ concentration and δ^18^O enrichment (Fig. 10).

Therefore, evaporation contributes to the increase in groundwater salinity, which is most visible downstream. A Cl^−^ vs. δ^18^O (Fig. 10) identifies the key mechanism controlling groundwater salinization. On the Fig. 10 a significant proportion of groundwater samples show a relatively stable δ^18^O with a higher chloride concentration than on the δ^18^O. The majority of the groundwater samples show mineral dissolution as the primary process. For the 2016 campaign, the Cl^−^ vs. δ^18^O relationship (Fig. 10a) shows that two groundwater samples (O3 and O4) had low chloride and δ^18^O values. This could mean groundwater is recharged in this area, especially in Meskala and Bouabout. This area is still characterized by the recharge zone in the 2020 campaign present at sample (EL 7) (Fig. 10b).

For the two campaigns, 2016 and 2020, two fundamental mechanisms leading to groundwater salinization in the examined aquifer were identified: (1) dissolution of evaporites, and (2) evaporation processes. In fact, the first step demonstrates a dissolving effect, with the isotopic compositions of the samples (δ^18^O) remaining unchanged as the chloride level increases. This model clearly supports the theory that the salinity of these waters is mainly controlled by dissolution.

For the 2016 and 2020 campaigns, two fundamental mechanisms leading to groundwater salinization in the aquifer under investigation were identified: (1) evaporite dissolution and (2) evaporation processes. The first class demonstrates a dissolution effect, with sample isotopic compositions (δ^18^O) remaining unchanged as chloride levels increase. This factor supports the theory that the salinity of these waters is primarily controlled by dissolution.

## Concluding remarks

Groundwater analysis is carried out using different methods and techniques to assess the usability of groundwater for irrigation and drinking purposes and the source of aquifer recharge. The water quality index, Piper plot, Wilcox plot, USSL plot, and isotopic stability (δ^18^O, δ^2^H) were used to demonstrate whether water samples from the Essaouira region are suitable for human use. Hydro-chemical facies reveal groundwater's nature is Na–Cl, mixed Ca–Mg–Cl and SO_4_–Ca type in the study area. The mean EC of the samples was determined 2453.8 μS/cm for the 2019 campaign, 2061.1 μS/cm for the for the 2020 campaign.

The results of water analyses were evaluated using the WQI and IWQI methods to assess groundwater quality for drinking and irrigation water use. Knowing the quality of groundwater in the region and determining the areas of use are important in terms of the sustainability of water management. According to the results obtained by the WQI method, not all groundwater samples in the study area are suitable for use as drinking water. In addition, according to the IWQI method, the groundwater samples are suitable for irrigation. In addition, using groundwater as drinking water may be hazardous to human health, and alternative drinking water resources should be investigated. On the other hand, to ensure the sustainable use of groundwater, it is necessary to control agricultural activities in the area, monitor the use of pesticides and fertilizers, and encourage organic farming practices.

The results suggest that groundwater consumption in the study area requires treatment and that most groundwater samples are in classes C3 and C4, which have the highest salinity and medium to high sodium risks, and can only be used on plants that tolerate high salinity.

The stable isotopic composition (δ^18^O and δ^2^H) of groundwater reveals that precipitation is the primary source of groundwater recharge and shows a higher depletion value in the recharge area than the discharge. In addition, the enriched isotopic composition δ^18^O > 5‰ indicates that groundwater is subjected to evaporation before recharge. This demonstrates that rainfall events result from the evaporation or mixing of elements with evaporated water before infiltration into the aquifer. In contrast, most points indicate the infiltration of water without evaporation. Atlantic precipitation continuously recharges watershed recharge areas between 2016 and 2020. The comprehensive approach to better understanding groundwater dynamics presented here could be a useful tool for managers to develop appropriate strategies for exploiting this resource.
